# Genotype-induced changes in biophysical properties of frontal cortex lipid raft from APP/PS1 transgenic mice

**DOI:** 10.3389/fphys.2012.00454

**Published:** 2012-11-27

**Authors:** Mario L. Diaz, Noemí Fabelo, Raquel Marín

**Affiliations:** ^1^Laboratorio de Fisiología y Biofísica de Membranas, Departamento de Biología Animal Facultad de Biología, Universidad de La LagunaTenerife, Spain; ^2^Departamento de Fisiología, Universidad de La LagunaTenerife, Spain

**Keywords:** lipid rafts, membrane viscosity, membrane thermodynamics, fluorescence anisotropy, cholesterol depletion, microdomain lipid composition

## Abstract

Alterations in the lipid composition of lipid rafts have been demonstrated both in human brain and transgenic mouse models, and it has been postulated that aberrant lipid composition in lipid rafts is partly responsible for neuronal degeneration. In order to assess the impact of lipid changes on lipid raft functional properties, we have aimed at determining relevant physicochemical modifications in lipid rafts purified from frontal cortex of wild type (WT) and APP/PS1 double transgenic mice. By means of steady-state fluorescence anisotropy analyses using two lipid soluble fluorescent probes, TMA-DPH (1-[(4-trimethyl-amino)phenyl]-6-phenyl-1,3,5-hexatriene) and DPH (1,6-diphenyl-1,3,5-hexatriene), we demonstrate that cortical lipid rafts from WT and APP/PS1 animals exhibit different biophysical behaviors, depending on genotype but also on age. Thus, aged APP/PS1 animals exhibited slightly more liquid-ordered lipid rafts than WT counterparts. Membrane microviscosity η_app_ analyses demonstrate that WT lipid rafts are more fluid than APP/PS1 animals of similar age, both at the aqueous interface and hydrophobic core of the membrane. η_app_ in APP/PS1 animals was higher for DPH than for TMA-DPH under similar experimental conditions, indicating that the internal core of the membrane is more viscous than the raft membrane at the aqueous interface. The most dramatic changes in biophysical properties of lipid rafts were observed when membrane cholesterol was depleted with methyl-β-cyclodextrin. Overall, our results indicate that APP/PS1 genotype strongly affects physicochemical properties of lipid raft. Such alterations appear not to be homogeneous across the raft membrane axis, but rather are more prominent at the membrane plane. These changes correlate with aberrant proportions of sphingomyelin, cholesterol, and saturated fatty acids, as well as polyunsaturated fatty acids, measured in lipid rafts from frontal cortex in this familial model of Alzheimer's Disease.

## Introduction

Although the existence of membrane rafts was disputed for many years, there is a general agreement that these specialized membrane nanodomains are specialized signaling platforms involved in a number of physiological functions under normal and pathological conditions (Allen et al., 2007; Michel and Bakovic, 2007). Nowadays, lipid rafts are defined as “… small (10–200 nm), heterogeneous, highly dynamic, sterol- and sphingolipid-enriched domains that compartmentalize cellular processes. Small rafts can sometimes be stabilized to form larger platforms through protein-protein and protein-lipid interactions” (Pike, 2006). From a biophysical point of view, rafts exist in viscous liquid-ordered state that phase segregates within the plasma membrane (Simons and Ikonen, 1997; Simons and Vaz, 2004).

Recent evidence has demonstrated that alteration of lipid raft homeostasis is linked to deregulation leading to neuronal loss in Alzheimer's disease. Alterations in these microdomains are known to affect amyloid precursor protein (APP) processing and neurotransmitter signaling (Rushworth and Hooper, 2006; Williamson and Sutherland, 2011; Hicks et al., 2012). Thus, lipid rafts play a central role in proteolytic processing and regulation of APP cleavage, and recent reports have shown that lipid rafts are the subcellular sites of amyloidogenic β-amyloid production by β-secretase 1 (BACE1) and the γ-secretase complex (Rushworth and Hooper, 2006; Williamson and Sutherland, 2011; Hicks et al., 2012). Further, both BACE1 and some γ-secretase subunits undergo post-translational S-palmitoylation which aids their targeting to lipid raft domains (Cheng et al., 2009). Further, recent findings have shown that lipid rafts act as key mediators of oxidative damage as a result of their ability to recruit Aβ-derived diffusible aggregates to the cell surface, triggering a further increase in membrane lipid peroxidation and loss of membrane integrity (Zampagni et al., 2010).

Double transgenic mice APP/PS1 expressing a chimeric mouse/human APP (Mo/HuAPP695swe: APP Swedish mutation) and a mutant human presenilin 1 (PS1-dE9), both directed to central nervous system neurons, have been generated as animal models of familial Alzheimer's disease. These animals (APP/PS1 mice) develop β-amyloid deposits throughout the brain and exhibit memory impairment earlier than single transgenic littermates. We have recently reported that this transgenic model show impaired memory and learning performance from the age of 6 months, which concurs with increased amyloid deposition, dystrophic neurites surrounding the plaques, altered expression of synaptic proteins, and malfunctioning of subcellular degradation pathways in the neorcortex (Aso et al., 2012). More recently, we have further explored the time-dependent alteration in lipid structure of neocortex. The outcomes revealed important age-dependent alterations in the lipid matrix of both cortical tissue and lipid rafts in APP/PS1 transgenic mice compared to wild type (WT) littermates, which were evident from the age of 6 months. Interestingly, changes in the lipid structure of lipid rafts could also be observed in the oldest WT group, whereby it was concluded that the AD genotype accelerates the normal aging process, provoking premature lipid raft aging (Fabelo et al., 2012b). Alteration of membrane lipids is expected to affect membrane biophysical properties, lipid-protein interactions, and membrane protein activity (Vigh et al., 2005). For a number of membrane proteins a modulatory effect has been demonstrated for membrane fluidity on protein activity, often affecting the thermodynamic properties of protein and lipid function (Almansa et al., 2003; Vigh et al., 2005; Tamm, 2006).

Therefore, in the present study we have aimed to determine the extent to which lipid changes in lipid rafts from APP/PS1 affect biophysical properties of the raft lipid microenvironment. It can envisaged that these changes may influence membrane associated proteins involved in amyloidogenic processing of APP.

## Materials and methods

### Isolation of lipid rafts

All experimental manipulations were performed following the procedures authorized by the Ethics Committee for manipulation of laboratory animals at University of La Laguna (Spain). WT and APP/PS1 mice were aged to 6 and 14 months and then sacrificed using carbon dioxide. The brains were dissected out on a chilled tray, and the frontal cortices rapidly isolated and collected in liquid nitrogen until lipid raft purification. At least four animals were analyzed in each age group from each genotype. Lipid raft fractions were isolated following the protocols described in detail in Fabelo et al. (2012a). Briefly, 0.1 g of brain cortex was homogenized at 4°C in isolation buffer (IB: 50 mM Tris-HCl, pH 8.0, 10 mM MgCl_2_, and 0.15 M NaCl) containing 1% Triton X-100 and 5% glycerol, 20 mM NaF, 1 mM Na_3_VO_4_, 5 mM β-mercaptoethanol, 1 mM PMSF, and a cocktail of protease inhibitors (Roche Diagnostics, Barcelona, Spain) in a glass homogenizer for 5 min, centrifuged at 500 g for another 5 min, and then the supernatant was collected and mixed in an orbital rotor for 1 h at 4°C. About 800 μL of the supernatant was mixed with an equal volume of 80% sucrose in IB and overlaid with 7.5 mL of a 36% sucrose solution and 2.7 mL of a 15% sucrose solution in IB, in 13.2 mL ultracentrifuge tubes (Ultraclear, Beckman). Sucrose gradients were centrifuged at 150,000 g for 18 h at 4°C using a Beckman SW41Ti rotor. Two mL fractions were collected from the top to the bottom and resuspended in 2 mL of IB. The first 2 mL fractions, contained the lipid rafts fractions, and were routinely tested for purity in Western blot assays using different lipid raft and non-raft protein markers (i.e., anti-flotillin 1 and anti-caveolin-1 for lipid rafts, anti-α1 subunit of the Na^+^/K^+^ ATPase for non-raft plasma membrane, and anti-Hsp90 for cytosolic proteins) following Fabelo et al. (2012b).

### Steady-state fluorescence anisotropy of lipid rafts

Steady-state anisotropy (*r*) measurements were performed using two different fluorescent probes: non-polar 1,6-diphenyl-1,3,5-hexatriene (DPH) and polar 1-[4-(trimethylammonio)phenyl]-6-phenyl-1,3,5-hexatriene (TMA-DPH). Lyophilized DPH and TMA-DPH were dissolved in 1 tetrahydrofuran:1 ethanol (v/v) and stored as 200 μM stock solutions. Probes were used at 2 μM in TBS (20 mM Tris–HCl buffer, pH 7.5, containing 150 mM KCl) prepared daily and stored protected from light until used. Lipid raft suspensions (250 μL, 50 μg protein/mL) were incubated in 1 mL vials at 37°C in PBS labeled solutions under agitation. Our preliminary data have shown that fluorescence anisotropy reached a constant value within approximately 10 min of labeling. Therefore, the results reported below were carried out following 30 min incubation with the probes. Fluorescence anisotropy values were determined in an Appliskan multiplate reader (Thermo Scientific) using 355 nm excitation filter and 420 nm emission filter, all equipped with polarizers. Controls containing the fluorophores alone were concurrently examined to correct for light scattering and intrinsic fluorescence. Fluorescence anisotropy for DPH and TMA-DPH were determined as follows:

$$r=\frac{I_{VV}-GI_{VH}}{I_{VV}+2GI_{VH}}$$

where *I*_*VV*_ and *I*_*VH*_ indicate the emitted fluorescence intensities in the vertical and horizontal planes, respectively, when excitation radiation is polarized vertically. *G* denotes the grating factor, a parameter determined for the instrument and given by *I*_*HV*_/*I*_*HH*_. Steady-state fluorescence anisotropy was initially determined at 38°C (the standard mouse body temperature). Afterwards, the same samples were cooled down to 20°C in a Peltier chamber and the fluorescence anisotropy measured against a ramp of temperatures from 20°C to 40°C.

Lipid rafts samples were also submitted to cholesterol depletion by methyl-beta-cyclodextrine (Mβ CD). For these experiments, lipid rafts were preincubated in TBS in the presence (Mβ CD group) or absence (control group) of 20 mM Mβ CD at 37°C for 1 h, before incorporation of the fluorescent probes.

### Lipid analyses

Lipid analyses were performed following standard procedures set up in our laboratory (Almansa et al., 2003; Martín et al., 2006). Briefly, total lipids from lipid rafts were extracted with chloroform/methanol (2:1 v/v) containing 0.01% of butylated hydroxytoluene (BHT) as antioxidant. Lipid classes were separated by one-dimensional double development high performance thin layer chromatography (HPTLC) using methyl acetate/isopropanol/chloroform/methanol/KCl 0.25% (5:5:5:2:1.8 v/v) as the developing solvent system for the polar lipid classes, and hexane/diethyl ether/acetic acid (22.5:2.5:0.25 v/v) as the developing solvent system for the neutral lipid classes. Lipid classes were quantified by charring with 3% (w/v) aqueous cupric acetate containing 8% (v/v) phosphoric acid a Shimadzu CS-9001PC dual wavelength flying spot scanner. Fatty acid methyl esters (FAME) from lipid rafts were obtained by acid-catalyzed transmethylation of total lipids for 16 h at 50°C, using 1 ml of toluene and 2 ml of 1% sulfuric acid (v/v) in methanol. The resultant FAME were purified by TLC and determined by reference to a conventional standard visualized under spraying with 1% iodine in chloroform. FAME were separated and quantified by using a TRACE GC Ultra (THERMO) gas chromatograph equipped with a flame ionization detector. Helium was used as a carrier gas.

### Statistics and calculations

Microviscosities (η) were derived as described previously for DPH (Laat et al., 1977) and TMA-DPH (Chazotte, 1994) by the method based on the Perrin equation for rotational depolarization of a non-spherical fluorophore:

$$\begin{array}{cc} \frac{r_{0}}{r}=1+C(r)\frac{T\tau }{\eta }\;(1)\Rightarrow \frac{C(r)\times \tau }{\left(\frac{r_{0}}{r}-1\right)}=\eta \times \frac{1}{T} & \left(2\right) \end{array}$$

where *r*_0_ and *r* are limiting and measured fluorescence anisotropies, *T* is the absolute temperature, and τ is the excited state lifetime. Limiting anisotropy (*r*_0_) for DPH and TMA-DPH was 0.395 (Prendergast et al., 1981). *C*(*r*) is a molecular shape parameter equal to 8.6 × 10^5^ poise·deg^−1^ s^−1^ and 15.3 × 10^5^ poise·deg^−1^ s^−1^ for DPH and TMA-DPH, respectively (Laat et al., 1977; Chazotte, 1994). Values of τ used were 10 and 7 ns for DPH and TMA-DPH, respectively. Microviscosity values were estimated from the slopes of $\theta \times {\left(\frac{r_{0}}{r}-1\right)}^{-1}$ *vs.* 1/*T* from Equation (2), being θ = *C*(*r*) × τ, and referred to as apparent microviscosity (η_app_). It should be stated that stated that the microviscosity values estimated here using constant probes' excited state lifetimes, provide only a quantitative approximation to real membrane microviscosity, since τ is known to vary with temperature and the membrane lipid system (Lakowicz, 1999). Discontinuity breakpoints (*Td*) were computed from Arrhenius representations. Data from lipid analyses were submitted to One-Way ANOVA followed by Tukey's *post-hoc* test or Student's *t*-test. Regression analyses were performed by non-linear regression using Sigmaplot software (Jandel Scientific, San Rafael, CA).

## Results

**Figure 1:**
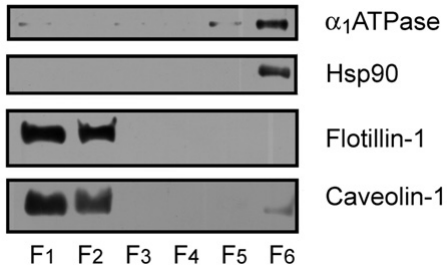
**Identification of protein markers in mouse cortex lipid raft by immunoblotting.** The six raft and non-raft fractions obtained by sucrose gradients were loaded on 10% SDS-PAGE and processed for Western blotting, using specific antibodies directed to the different protein markers. Lipid raft resident proteins flotilllin-1 and caveolin-1 were abundantly detected in raft fractions 1 and 2, whereas the α 1 subunit of Na^+^/K^+^ ATPase (α 1ATPase), and the cytosolic protein Hsp90 were detected in non-raft fraction 6.

We have assayed the steady-state fluorescence anisotropy of purified lipid raft samples from frontal cortex of WT and APP/PS1 animals at two different ages: 6 months and 14 months. These two ages were chosen according to our previous results demonstrating the existence of differences in the lipid structure of lipid rafts between the two genotypes (Fabelo et al., 2012b). Results in Figure 1 shows that isolated membranes comply with the biochemical standards for lipid rafts, i.e., expression of the specific scaffolding protein markers flotilin-1 and caveolin-1 (Lucero and Robbins, 2004), exclude non-raft marker Na^+^/K^+^ ATPase, and exhibit high levels of cholesterol, sphyngomyelin, and saturated fatty acids contents (Table 1). We used two different probes, the non-polar probe DPH to determine the physical order at the hydrophobic core of the lipid raft membrane and the polar TMA-DPH to assess rotational freedom at the membrane surface (Lentz, 1993). For structural purposes, we present the results for each probe separately and, at the end of this section, we described the results of microviscosity-lipid relationships.

**Table 1:** **Lipid classes and indexes in lipid rafts from isolated from frontal cortex of WT and APP/PS1 aged 6 and 14 months**.

	**WT 6 months**	**APP 6 months**	**WT 14 months**	**APP 14 months**
Sphingomyelin (SM)^1^	3.17 ± 0.15^c^	4.71 ± 0.34^***^^c^	6.05 ± 0.81	7.25 ± 0.47
Phosphatidylcholine (PC)^1^	11.62 ± 1.37^a^	12.27 ± 0.18^b^	15.58 ± 0.45	15.85 ± 0.78
Cholesterol (CHO)^1^	31.88 ± 4.75	29.40 ± 1.01^c^	25.94 ± 0.47	22.97 ± 0.95^**^
SM/PC	0.30 ± 0.05	0.48 ± 0.03^**^	0.40 ± 0.06	0.46 ± 0.05
SM/CHO	0.11 ± 0.01^c^	0.16 ± 0.02^c^	0.24 ± 0.03	0.32 ± 0.02^*^
Saturated^2^	58.45 ± 0.92^c^	59.95 ± 0.14^c^	68.15 ± 1.46	71.89 ± 1.01^*^
Unsaturated^2^	38.25 ± 2.20^b^	31.05 ± 2.48^*^	31.03 ± 1.32	27.45 ± 1.04^*^
Monoenes^2^	20.65 ± 1.41	17.81 ± 1.78	18.46 ± 0.65	18.46 ± 1.05
LCPUFA^2^	16.72 ± 2.04^a^	11.90 ± 0.63^b^	12.27 ± 0.90	8.66 ± 0.65^***^
Total n-9^2^	13.36 ± 1.22^a^	10.02 ± 1.91	10.73 ± 0.68	9.80 ± 0.82
Total n-6^2^	8.93 ± 0.57^c^	7.71 ± 0.35^c^	6.45 ± 0.44	4.92 ± 0.35^**^
Total n-3^2^	8.49 ± 1.38^a^	5.47 ± 0.36^b^	5.92 ± 0.53	3.78 ± 0.32^***^
saturated/unsaturated	1.56 ± 0.13^c^	1.97 ± 0.16^*^^b^	2.23 ± 0.14	2.64 ± 0.15^*^
saturated/n-3	7.75 ± 1.38^b^	11.11 ± 0.76^c^	12.07 ± 1.28	19.50 ± 1.46^***^
saturated/n-6	6.67 ± 0.50^c^	7.80 ± 0.37^c^	10.87 ± 0.92	14.90 ± 1.05^**^
saturated/n-9	4.57 ± 0.54^b^	6.72 ± 1.29	6.53 ± 0.58	7.63 ± 0.90
saturated/CHO	1.99 ± 0.27^a^	2.04 ± 0.07^c^	2.63 ± 0.07	3.15 ± 0.11^***^
SM/unsaturated	0.08 ± 0.01^c^	0.15 ± 0.00^***^^c^	0.20 ± 0.03	0.26 ± 0.02
SM/n-3 LCPUFA	0.41 ± 0.06^c^	0.86 ± 0.00^***^^c^	1.08 ± 0.16	1.95 ± 0.14^***^
CHO/unsaturated	0.83 ± 0.10	0.96 ± 0.11	0.84 ± 0.04	0.85 ± 0.07
CHO/LCPUFA	1.90 ± 0.13	2.49 ± 0.22^**^	2.17 ± 0.17	2.69 ± 0.15^*^
Phospholipids/Cholesterol	1.81 ± 0.35	1.78 ± 0.09^c^	2.22 ± 0.07	2.61 ± 0.11^**^
Unsaturation index	105.99 ± 10.72^b^	78.91 ± 5.18^b^	79.32 ± 5.04	60.40 ± 3.18^**^

Data are expressed mean ± SEM for 4–5 animals in each group of genotype and age.

1 Weight percent respect to total lipids from lipid raft,

2 weight percent respect to total fatty acids from lipid raft.

* ^,^

** ^,^

*** Indicate statistical differences with p = 0.1, p = 0.05 and p = 0.01 between genotypes of same age.

a ^,^

b ^,^

c Indicate statistical differences with p = 0.1, p = 0.05 and p = 0.01 between ages within each genotype.

### TMA-DPH

**Figure 2:**
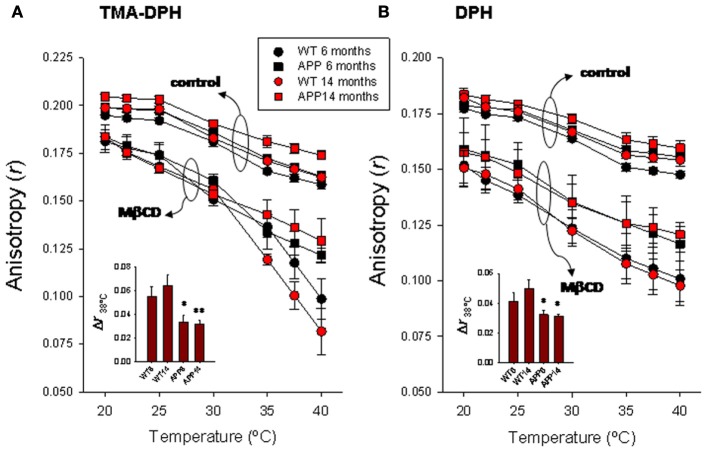
**Temperature-dependence of steady-state fluorescence anisotropy for TMA-DPH (A) and DPH (B) in frontal cortex lipid rafts.** Lipid rafts were isolated from WT and APP/PS1 animals aged up to 6 and 14 months. The insets summarize the results of Δ *r* determined at 38°C, the animal's body temperature. ^*,**^ *p* < 0.05 and *p* < 0.01 compared to WT animals of similar age.

Results summarized in Figure 2A show the TMA-DPH anisotropy values measured in the range 20–40°C in lipid rafts from wild-type and APP/PS1 animals aged to 6 and 14 months. Anisotropy values of lipid rafts decreased with temperature in all groups both under control conditions or treated with methyl-β-cyclodextrin (Mβ CD). Under control conditions, anisotropy values were slightly larger in 14 months old (mo) APP/PS1 than in all other groups, especially in the range 35–40°C, where differences were statistically significant (*F* = 5.23, *p* < 0.01). Treatment with methyl-β-cyclodextrin brings about a considerable reduction of lipid raft fluorescence anisotropy in all groups compared with controls, but the effect was notably more important in WT animals. The preincubation protocol with Mβ CD used here (20 mM for 1 h), causes a ~50% depletion of membrane cholesterol (Zidovetzki and Levitan, 2007). As a result of cholesterol reduction, anisotropy difference (Δ*r*) determined at 38°C (the animals' body temperature) was 1.66–2.04 times larger in WT than in APP/PS1 animals of similar age (Figure 2A inset of left panel).

**Figure 3:**
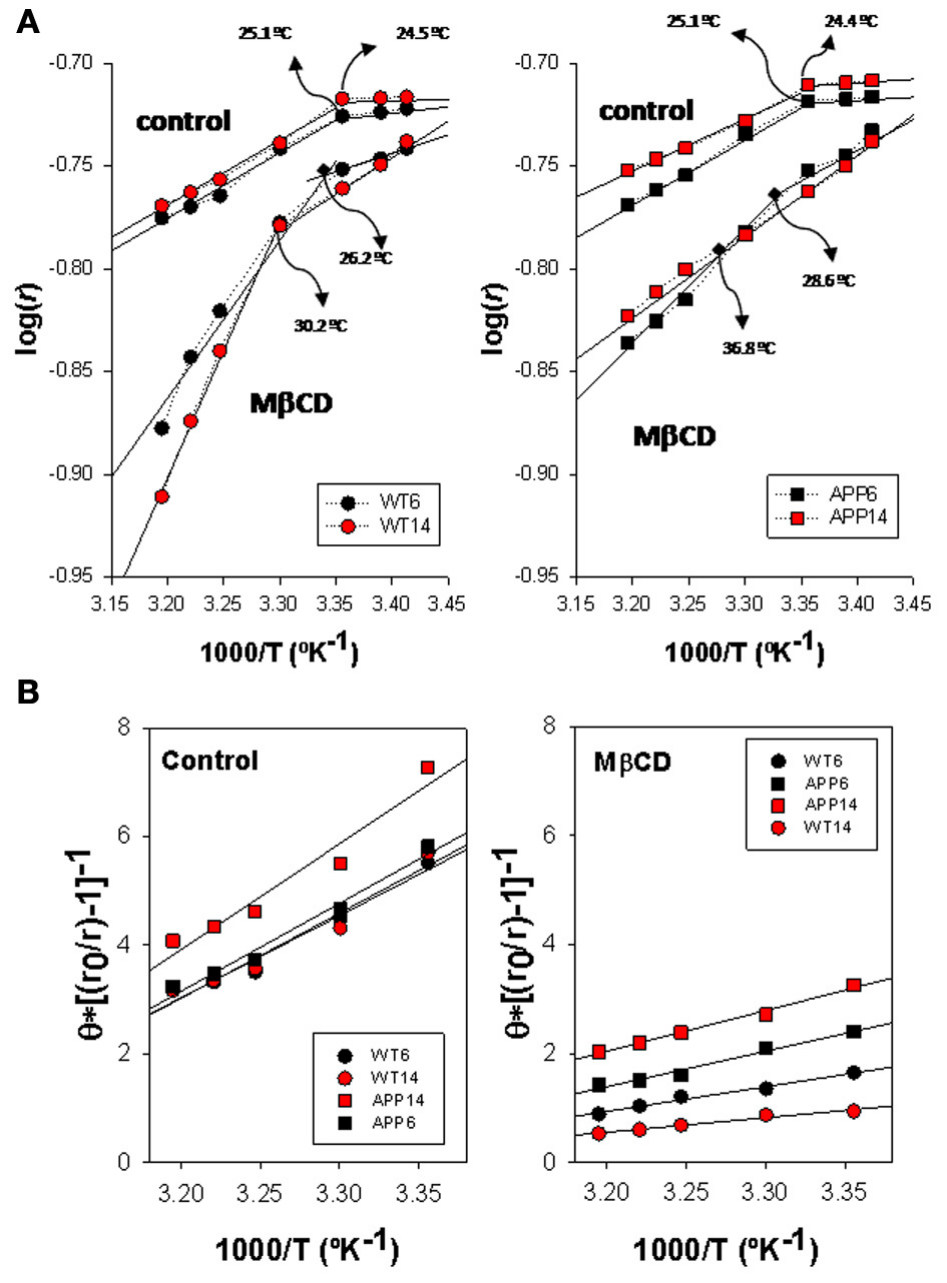
**Biophysical characterization of lipid rafts from AP/PS1 and WT brain cortex probed with TMA-DPH. (A)** Arrhenius plots for steady-state anisotropy in WT (left) and APP/PS1 (right) animals. **(B)** Microviscosity analyses based on modified Perrin equation for steady-state fluorescence anisotropy for TMA-DPH under control (left panel) and after Mβ CD treatment (right panel) temperature. Discontinuity temperatures (*Td*) are indicated.

Arrhenius plots for TMA-DPH anisotropy data from WT and APP/PS1 is shown in Figure 3A. Discontinuity points (*Td*) in the range 24.4–25.1°C can be identified for intact lipid rafts (controls) in both genotypes, indicating the existence of thermotropic phase transitions. No differences in *Td* were observed between lipid rafts from WT (left panel) and APP/PS1 (right panel) animals. This homogeneity in control lipid rafts from WT and APP/PS1 was markedly broken upon cholesterol depletion by Mβ CD (Zidovetzki and Levitan, 2007). Thus, in WT animals, Mβ CD treatment shifted *Td* values toward higher temperatures (up to 30.2°C for 14 mo mice), but the displacement of *Td* was notably more dramatic in APP/PS1 animals, up to 28.6°C and 36.8°C for 6- and 14-months mice, respectively.

Using the modified expression of Perrin Equation shown in (2), we have assessed the changes in lipid raft microviscosity in the different animals and conditions (Figure 3B). Under control conditions, microviscosity values in WT animals were nearly the same between 6 and 14 mo animals (15.13 poises and 15.66 poises, respectively). In APP/PS1 animals, while in the youngest animals the microviscosity value (16.21 poises) was similar to WT animals, a notable increase was observed for 14 mo animals (19.5 poises), indicating a clear influence of age on the APP genotype. Upon cholesterol depletion, apparent microviscosity values were dramatically reduced to 3.62 and 2.49 poises in 6 and 14 mo WT mice, these values representing a reduction of about 80% compared to control conditions. On the other hand, in transgenic animals, lipid rafts microviscosity values following Mβ CD treatment (6.12 and 9.26 poises, for 6 and 14 mo WT mice, respectively), remained significantly higher than in WT animals of same ages. Thus, Mβ CD treatment decreased lipid raft microviscosity by average 55% compared to control conditions, substantially lower than in WT animals.

### DPH

DPH anisotropy in lipid rafts (Figure 2B right panel) followed a similar pattern to that observed for TMA-DPH. Under control conditions, highest anisotropy values were observed for 14 mo APP/PS1 animals, while the lowest values were seen in 6 mo WT animals. Anisotropy values were nearly identical between youngest APP/PS1 and oldest WT animals. Treatment with Mβ CD decreases anisotropy in all groups, and again the reduction was substantially greater in WT than in APP/PS1 animals at all temperatures assayed. Consequently, Δ*r*_38°C_ was significantly larger in WT animals compared to APP/PS1 animals (Figure 2B inset of right panel). However, unlike TMA-DPH, changes in APP/PS1 animals and WT lipid rafts in the presence of Mβ CD appeared not to be affected by age, since anisotropy values were nearly identical in 6 and 14 mo, suggesting that anisotropy changes are exclusively related to the AD genotype.

**Figure 4:**
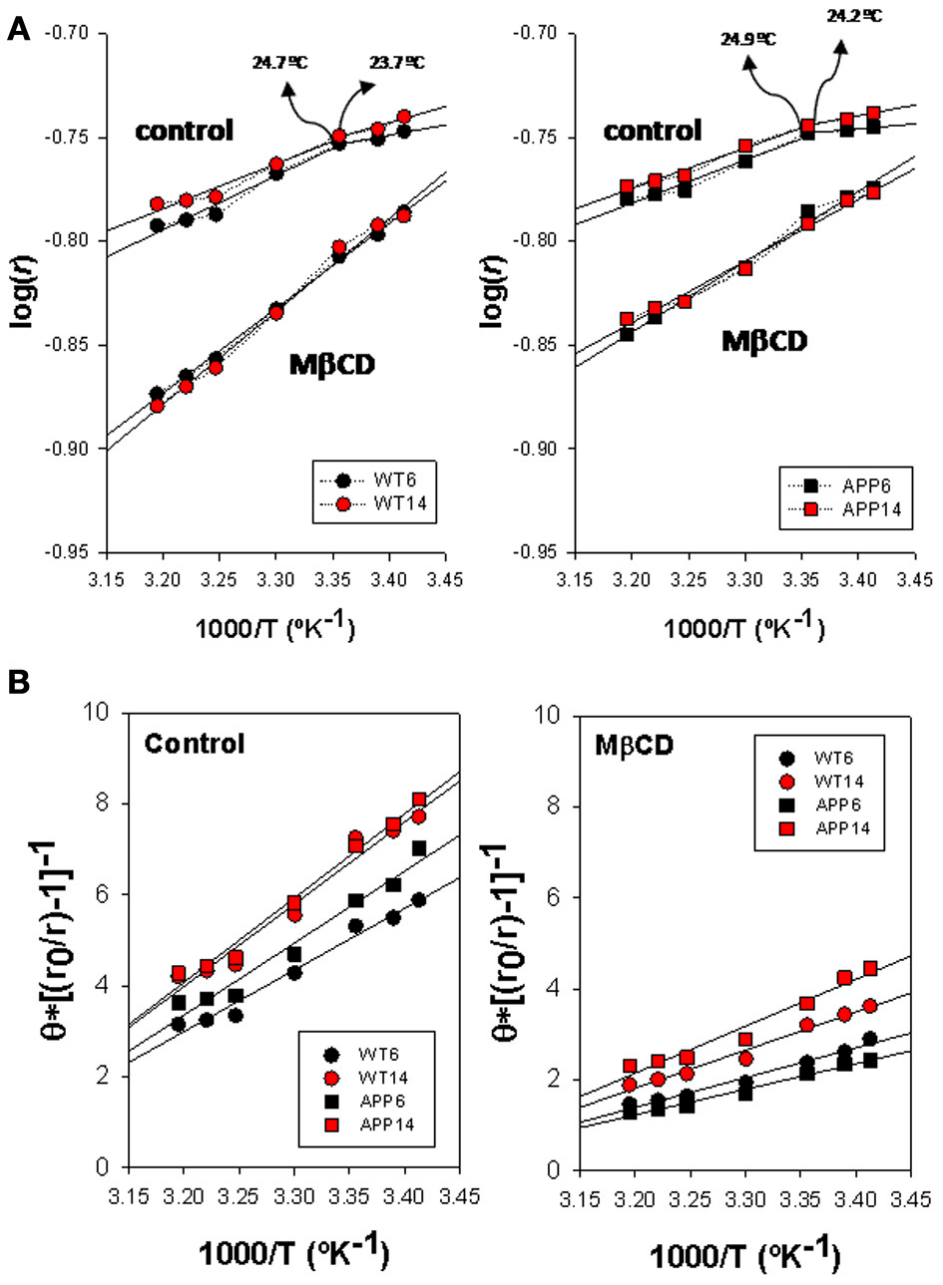
**Biophysical characterization of lipid rafts from APP/PS1 and WT brain cortex probed with DPH. (A)** Arrhenius plots for steady-state anisotropy in WT (left) and APP/PS1 (right) animals. **(B)** Microviscosity analyses based on modified Perrin equation for steady-state fluorescence anisotropy for DPH under control (left panel) and after Mβ CD treatment (right panel). Discontinuity temperatures (*Td*) are indicated.

Arrhenius breakpoints are also evident in DPH fluorescence anisotropy measurements in WT (Figure 4A, left panel) and APP/PS1 (Figure 4A, right panel) lipid rafts, being *Td* values under control conditions very close to those obtained for TMA-DPH. Strikingly, in the presence of Mβ CD it was not possible to identify any discontinuity point neither in WT nor in APP/PS1 animals. Vanishing of *Td* values in the presence of critically low levels of cholesterol, indicate that the cholesterol depleted lipid raft environment hampers phase transitions at the hydrophobic core.

η_app_ values (Figure 4B) in control membranes were significantly lower in WT than in APP/PS1 lipid rafts (13.98 poises vs. 19.21 poises, in 6 mo WT and APP/PS1 animals, respectively, and 14.36 poises vs. 18.07 poises in 14 mo WT and APP/PS1 animals, respectively), indicating that lipid rafts in transgenic animals are ~20–30% more viscous than WT littermates. Within each genotype, age did not affect microviscosity, suggesting that augmented η_app_ values in APP/PS1 animals is a characteristic of lipid rafts from transgenic animals. A similar conclusion was reached in cholesterol depleted lipid raft, i.e., lower η_app_ in APP/PS1 than in WT animals. Further, the decrease in membrane raft microviscosity was higher in WT (η_app_ values of 7.60 and 6.54 poises, for 6 and 14 mo) compared to APP/PS1 animals (η_app_ values of 9.73 and 11.99 poises, for 6 and 14 mo), representing increases of 56.4% and 60.8% in 6 and 14 mo, respectively) compared to transgenic littermates (47.3%. and 42.9% in 6 and 14 mo, respectively).

### Lipid characteristics of lipid rafts

We have recently reported the effects of APP/PS1 genotype on lipid profiles from frontal cortex lipid rafts along age (Fabelo et al., 2012b). In this study we have extended the determinations in 6 and 14 mo animals to assess relevant lipid classes (sphingomyelin, cholesterol, phosphatidylcholine, saturated fatty acids, unsaturated fatty acids, LCPUFA, n-3, n-6, n-9, and monoenes), and lipid indexes (unsaturation index, saturated/unsaturated, phospholipids/cholesterol, sphingomyelin/cholesterol, sphingomyelin/phosphatidylcholine, sphingomyelin/n-3 LCPUFA, saturated/n-6, saturated/n-3 LCPUFA, and saturated/n-9, CHO/LCPUFA) as potential biochemical correlates of biophysical changes. Results in Table 1 shows that APP/PS1 genotype alters lipid signature of frontal cortex lipid rafts compared with WT both at 6 and 14 mo. Thus, at the age of 6 mo, lipid rafts from APP/PS1 animals exhibit higher levels of sphingolmyelin (SM) and lower contents of unsaturated fatty acids compared to WT animals. Consequently, saturated/unsaturated, cholesterol/LCPUFA, sphyngomyelin/unsaturated, and sphyngomyelin/n-3 LCPUFA ratios were all augmented in transgenic mice. Changes were more dramatic in APP/PS1 animals at the age of 14 months. These animals showed higher contents of saturates and lower levels of cholesterol, unsaturated fatty acids, LCPUFA, n-6, and n-3 than WT animals of same age. The ratios SM/CHO, saturated/unsaturated, saturated/n-3 LCPUFA, saturated/CHO, SM/n-3 LCPUFA, SM/unsaturated, and phospholipids/cholesterol were all significantly increased compared to WT lipid rafts. Overall these changes indicate that lipid rafts from APP/PS1 animals are considerably more saturated, enriched in SM and impoverished in cholesterol compared to age matched WT animals.

**Figure 5:**
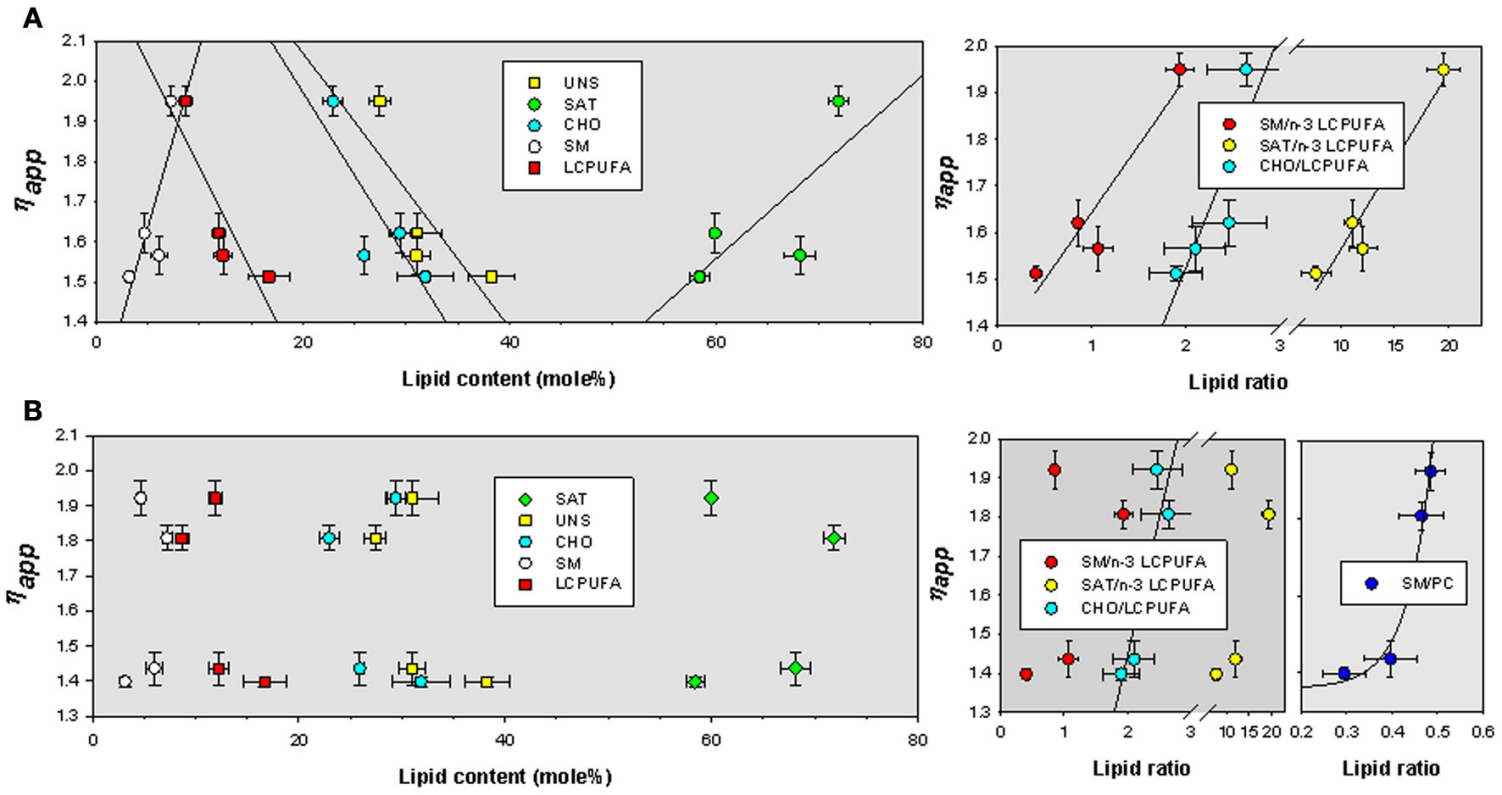
**Bivariate relationships for η_app_ as a function of lipid classes and lipid indexes for TMA-DPH (A) and DPH (B) in lipid rafts isolated from APP/PS1 and WT animals.** SAT, total saturates; UNS, total unsaturated; CHO, cholesterol; SM, sphingomyelin; LCPUFA, long chain polyunsaturated fatty acids; n-3 LCPUFA, total n-3 LCPUFA. PC, phosphatidylcholine.

We have finally assessed the quantitative relationships between lipid indicators and biophysical parameters. We have used multiple correlation analyses to extract bivariate relationships displaying determination coefficients above 0.7. The values were plotted and fitted to different lineal and exponential functions by non-linear regression. For TMA-DPH microviscosity values were positively related to SM and saturates, and negatively to cholesterol, unsaturated and, very significantly, to LCPUFA (Figure 5A, left panel). The best descriptive parameters in determining η_app_ for TMA-DPH were the ratio saturated/n-3 LCPUFA (*R*^2^ = 0.93), SM/n-3 LCPUFA (*R*^2^ = 0.89), and CHO/LCPUFA (*R*^2^ = 0.74) (Figure 5A, right panel). Upon cholesterol depletion, η_app_ was mainly related to saturates (*R*^2^ = 0.88) and the ratios SM/PC (*R*^2^ = 0.80) and saturated/unsaturated (*R*^2^ = 0.72) (not shown). Strikingly, we found that η_app_ for DPH was mostly unrelated to lipid indicators (Figure 5B left panel), except for CHO/LCPUFA that was linearly correlated (*R*^2^ = 0.80) and SM/PC that was exponentially related to η_app_ (*R*^2^ = 0.97) (Figure 5B, rightmost panel).

## Discussion

In the present study, we have assessed the impact of lipid changes in lipid raft isolated from transgenic and wild-type frontal cortex animals on lipid raft biophysical properties using steady-state fluorescence anisotropy analyses (Lentz, 1993). We have observed that lipid rafts from aged APP/PS1 animals exhibited higher TMA-DPH and DPH anisotropy values than all the other groups, and that 6 mo WT animals showed the lowest values. These differences were more evident around the physiological temperature of 38°C. Such an increase in membrane order in APP/PS1 cannot be explained by the reduced membrane cholesterol observed in this study, since reduction of cholesterol would be expected to increase fluidity (Van Blitterswijk et al., 1987; Spink et al., 1996), especially at the high temperatures of endothermic animals. Cholesterol interacts with membrane phospholipids and sphingolipids, and modulates membrane physical behavior (Ohvo-Rekilä et al., 2002). Thus, incorporation of increasing levels of cholesterol broadens, and eventually abolishes, the cooperative gel/liquid-crystalline phase transition of the host lipid bilayer (Lewis and McElhaney, 1992). Cholesterol induces an “intermediate state” in phospholipid molecules with which it interacts, increasing the fluidity of the hydrocarbon chains below the *Tm* (gel to liquid-crystalline phase transition temperature) but decreases the fluidity above it (Finegold, 1993; Maulik and Shipley, 1996). In the biologically relevant liquid-crystalline state, cholesterol increases the degree of orientational order, reduces the rate of motion of the phospholipid hydrocarbon chains, and lead to a laterally more condensed membrane, with increased packing density of the phospholipids (Finegold, 1993; Ohvo-Rekilä et al., 2002). Reduction of cholesterol mass or cholesterol partition coefficient causes the opposite effects in membranes (Tsamaloukas et al., 2005; Zidovetzki and Levitan, 2007). In agreement with this, we observed that induction of cholesterol depletion by membrane cholesterol extraction with Mβ CD provokes the expected reduction of fluorescence anisotropy and membrane order (Gidwani et al., 2001; Zidovetzki and Levitan, 2007; Jiang et al., 2010). Interestingly, the magnitude of the change in Δ*r*_38°C_ is considerably larger in WT animals than in APP/PS1 animals, which is consistent with the greater amount of cholesterol in lipid rafts from WT animals (Fabelo et al., 2012b).

By comparing the effects of the polar and non-polar lipid-soluble probes used here, it may be concluded that the higher membrane order of lipid rafts in APP/PS1 was due to changes in the lateral mobility of lipids within the membrane plane, rather than in the hydrophobic core of the bilayer (Lentz, 1993; Kaiser and London, 1998). This observation indicates a greater degree of packing of membrane phospholipids, which may provide more abundant hydrophobic interactions between phospholipid fatty acids in the membrane plane. A deeper insight into the differences of the physicochemical status of lipid rafts from WT and APP/PS1 was obtained from the temperature-dependence analyses of fluorescence anisotropy. First, we observed clear discontinuity points around 24.5°C separating the more ordered gel and liquid crystalline disordered stages. These rather low temperature breakpoints were similar between ages and genotypes but were strongly sensitive to cholesterol depletion. Thus, Mβ CD shifted *Td* toward higher temperatures in the TMA-DPH analyses and virtually made it disappear for DPH, indicating the critical role of cholesterol in determining phase transition behavior, which might be, at least in part, driven by enthalpy changes. In agreement, using differential scanning calorimetry and x-ray diffraction to investigate the interaction of N-stearoylsphingomyelin with cholesterol and dipalmitoylphosphatidylcholine, Maulik and Shipley (1996) have shown that fully hydrated C18:0-SM forms bilayers that undergo a gel—liquid-crystalline transition at 45°C with Δ *H* 6.7 kcal/mol but addition of cholesterol results in a progressive decrease in the enthalpy of the transition at 45°C. Further, disruption of the gel phase by altering the partition coefficients for cholesterol in phospholipid bilayers, increase enthalpies of transfer to liquid phase containing di-C18-C20 phosphatidylcholine, and this energetic phenomenon is accompanied by a compensating increase in the entropy of transfer (Tsamaloukas et al., 2005). Obviously removal of cholesterol forces remaining lipid species to readapt in the lipid matrix. The physical consequences of such energetic pressure is that most abundant sphyngolipids and saturated phospholipids must re-accommodate in the presence of significant amounts of unsaturated fatty acids in the membrane plane, being larger the activation enthalpy the more abundant cholesterol was in the native membrane.

Apart from cholesterol, APP/PS1 lipid rafts also contained significantly increased levels of sphingomyelin and phosphatidylcholine compared to 6 mo animals. It is known that these two lipid classes promote membrane packaging through hydrophobic interactions between their acyl chains and also with the planar rings of cholesterol favoring phase separation of liquid order domains (Slotte, 1999; Ohvo-Rekilä et al., 2002). A considerable amount of data indicates that cholesterol interacts more favorably with sphingomyelin than with phosphatidylcholine, in both bilayer and monolayer membranes (Slotte, 1999; Ohvo-Rekilä et al., 2002; Simons and Vaz, 2004). Perhaps the most striking difference between sphingomyelin and phosphatidylcholine is the structural dissimilarity of chain length in SM, where the *N*-acyl group (usually highly saturated) can be up to 10 carbons longer than the sphingosine (Barenholz and Thompson, 1980). Such a chain disparity give SMs the unique ability to form both intra- and inter-molecular hydrogen bonding and increases the hydrophobic interaction with other membrane components within the membrane plane, but also enables sphingomyelins to participate in transbilayer hydrocarbon interdigitation (Slotte, 1999; Ohvo-Rekilä et al., 2002; Sonnino and Prinetti, 2010).

The increase in SM and PC contents measured here, on their own, might explain the increased membrane order observed in aged animals. However, we have observed that levels of these two lipid classes were similarly augmented in oldest WT and APP/PS1 animals. We also detected a significant increase in the SM/CHO ratio not only between ages but also between genotypes (oldest APP/PS1 animals displayed the largest SM/CHO ratio and youngest WT animals the lowest values), but the analyses revealed no statistically significant quantitative relationships between these lipid classes or indexes (SM/CHO, SM/PC, and PHO/CHO), and membrane order parameters in control animals. Therefore, we thought differences in the thermodynamic behavior of lipid rafts between WT and APP/PS1 might have been attributable to differences in the proportions of a different group of fatty acids rather than lipid classes, namely saturated and unsaturated fatty acids, or relative proportions between lipid classes to subgroups of fatty acids. We have measured significant amounts of n-9, n-6, and n-3 unsaturated fatty acids in lipid raft from both WT and APP/PS1 animals (Fabelo et al., 2012b), and also in human cortex from control and AD donors (Martín et al., 2010; Fabelo et al., 2011). We report here that aging is accompanied by increased proportions of saturated fatty acids and an important reduction of n-6 and n-3 long chain polyunsaturated fatty acids. Notably, n-9 monounsaturated fatty acids were unchanged. These changes have previously described in detail by our group and gave rise to the notion that lipid rafts are subjected to an aging process (lipid raft aging hypothesis) which is exacerbated by the presence of the APP/PS1 genotype (Fabelo et al., 2012b).

For long, lipid rafts have been biochemically defined by their high cholesterol, sphingomyelin, and saturated fatty acids levels, which provide a liquid ordered environment that phase segregate in the plasma membrane (Simons and Ikonen, 1997; Brown and London, 2000). Though it is accepted the extreme biochemical complexity of plasma membrane lipids, most information on the biophysical characteristics of lipid rafts domains derives from studies in model membranes. These artificial bilayers are made up of specific proportions of different constituents, mainly cholesterol, sphingomyelin, and phosphatidylcholine forming binary and ternary lipid systems, where the presence of unsaturated phospholipids favors phase segregation of raft-like structures (Slotte, 1999; Simons and Vaz, 2004). In lipid rafts from living cells though, long chain polyunsaturated phospholipids may be components of the lipid raft phase (Fridriksson et al., 1999; Stulnig et al., 2001; Pike et al., 2002; Corsetto et al., 2012). These lipids prefer the non-raft phase, and this may limit the domain size when the probability of encountering them in the raft domain perimeter exceeds a certain value (Simons and Vaz, 2004). In fact, several studies in cell lines have shown that LCPUFA enrichment, especially with n-3 PUFA, alters size, stability and distribution of lipid rafts (Stulnig et al., 2001; Corsetto et al., 2012), with the formation of declustered rafts (Shaikh, 2012). One major concern on the results from cell lines cultured under standard media is that plasma membrane lipids, including lipid rafts, exhibit a lipid profile that differs substantially from the cells they supposedly derive from. In particular cell lines are largely depleted in polyunsaturated fatty acids (Martín et al., 2006), a finding that may be attributable to the virtual absence of LCPUFA in culture media and the limited capacity to elongate LCPUFA from metabolic n-3 and n-6 precursors. This is particularly important for neuronal cell lines whose plasma membrane usually contain less than 1% n-3 LCPUFA (mainly docosahexaenoic acid, DHA) while in neural tissue these fatty acids are present in much larger amounts, between 10 and 20% (Carrié et al., 2000; Martín et al., 2006; Fabelo et al., 2012a,b). Obviously, differences in the lipid composition of lipid raft in cellular models compared to native tissues underestimate the physiological role of lipid classes present in minor proportions while blurs their significance in the biophysical properties of the membrane.

The impact of lipid changes on membrane lipid order in APP/PS1 and WT animals is reflected in membrane microviscosity. We observed that genotype substantially affect apparent microviscosity and, unexpectedly, that the degree of alteration depends on the deepness of the membrane considered. In the membrane plane, combined effects of aging and genotype lead to more viscous lipid rafts in APP/PS1 animals. Thus, at the physiological temperature of 38°C, lipid rafts microviscosity in the oldest animals is 25% higher in transgenic animals. Cholesterol depletion by Mβ CD treatment reduces exofacial microviscosity in lipid raft but still values remained significantly higher in APP/PS1 animals of both ages (−79% and −82% in WT6, WT14, respectively, versus −62% and −52% for APP/PS1 6 and 14 mo animals, respectively), indicating that it is the genotype (overexpression of amyloid beta) what determines the physical deviation of membrane viscosity in transgenic animals. This conclusion was reinforced by the observations at the interfacial level using DPH probe, where microviscosity values were always larger (average 32%) for lipid rafts from APP/PS1 than for WT animals at all ages. Apparent microviscosity was linearly related to lipid classes, particularly in the membrane plane. Thus, we observed positive relationships for saturates and sphingomyelin, and negative for cholesterol, unsaturated and very significantly for LCPUFA. However, the best predicting factors for TMA-DPH-related viscosity were the ratios saturated/n-3 LCPUFA, SM/n-3 LCPUFA, and CHO/LCPUFA, which were positively associated to η_app_. Thus, from these results it can be concluded that the increased viscosity of membrane leaflets is related to lipid species that increase membrane hydrophobic interactions and force membrane packaging (Ohvo-Rekilä et al., 2002; Simons and Vaz, 2004), but the net effect on membrane viscosity is determined by the presence of unsaturated fatty acids. This assertion explains why η_app_ values are higher than expected in 14 mo APP/PS1 transgenic mice, where cholesterol contents are slightly reduced (12%, and the expected effect would be an increase in fluidity) but those of LCPUFA to a much larger extent (30%). Besides, the larger reduction in lateral plane viscosity in the presence of Mβ CD in WT compared to APP/PS1 animals may be interpreted on the basis of the larger proportions of LCPUFA in WT mice that counteracts the lateral packaging effect of SM, which dominates in APP/PS1 mice (at the time that LCPUFA levels are reduced). Finally, it is worth mentioning that when examined at the hydrophobic core, APP/PS1 animals exhibit always larger microviscosity values than WT animals of either age. We show that in DPH assays, microviscosity values were less affected by LCPUFA than at the aqueous interface. In fact, no obvious relationships exist for gross lipid classes' contents or lipid indexes, except for the ratios CHO/PUFA and SM/PC, which related exponentially to η_app_. The latter relationship was mechanistically interesting since the saturated long acyl-chain and hydrophobic mismatch in SM respect to phosphatidylcholine, may enable transbilayer hydrocarbon interdigitations as it has been shown in lipid bilayers (Morrow et al., 1995; Li et al., 2001; Sonnino and Prinetti, 2010). This structural feature of SM (in the presence of reduced amounts LCPUFA in PC) likely provides additional hydrophobic interactions between leaflets, thereby positively affecting ordering at the hydrophobic membrane core. It could be envisaged that these changes in membrane transbilayer interactions might contribute to the anomalous thermotropic behavior of membrane rafts from APP/PS1 animals observed here.

In summary, we report here a mechanistic scenario for the consequences of alterations in the lipid matrix of lipid rafts on the physicochemical characteristics of these microdomains in APP/PS1 animals overexpressing amyloid beta peptides. We conclude that the APP/PS1 genotype, rather than age, is a crucial factor in determining the lipid structure of lipid rafts which, in turn, determines important biophysical alterations in these microdomains. At least in part, the abnormal physicochemical behavior of APP/PS1 lipid rafts is due to altered contents of LCPUFA. The extent to which these observations might concur in the human cortex in Alzheimer's disease is currently under study in our laboratory.

### Conflict of interest statement

The authors declare that the research was conducted in the absence of any commercial or financial relationships that could be construed as a potential conflict of interest.
